# Interpretable cardiac anatomy modeling using variational mesh autoencoders

**DOI:** 10.3389/fcvm.2022.983868

**Published:** 2022-12-22

**Authors:** Marcel Beetz, Jorge Corral Acero, Abhirup Banerjee, Ingo Eitel, Ernesto Zacur, Torben Lange, Thomas Stiermaier, Ruben Evertz, Sören J. Backhaus, Holger Thiele, Alfonso Bueno-Orovio, Pablo Lamata, Andreas Schuster, Vicente Grau

**Affiliations:** ^1^Institute of Biomedical Engineering, Department of Engineering Science, University of Oxford, Oxford, United Kingdom; ^2^Division of Cardiovascular Medicine, Radcliffe Department of Medicine, University of Oxford, Oxford, United Kingdom; ^3^University Heart Center Lübeck, Medical Clinic II, Cardiology, Angiology, and Intensive Care Medicine, Lübeck, Germany; ^4^University Hospital Schleswig-Holstein, Lübeck, Germany; ^5^German Centre for Cardiovascular Research, Partner Site Lübeck, Lübeck, Germany; ^6^Department of Cardiology and Pneumology, University Medical Center Göttingen, Georg-August University, Göttingen, Germany; ^7^German Centre for Cardiovascular Research, Partner Site Göttingen, Göttingen, Germany; ^8^Department of Internal Medicine/Cardiology, Heart Center Leipzig at University of Leipzig, Leipzig, Germany; ^9^Leipzig Heart Institute, Leipzig, Germany; ^10^Department of Computer Science, University of Oxford, Oxford, United Kingdom; ^11^Department of Biomedical Engineering, King's College London, London, United Kingdom

**Keywords:** mesh VAE, 3D ventricular shape analysis, virtual anatomy generation, clinical outcome prediction, acute myocardial infarction, major adverse cardiac events, graph neural networks, geometric deep learning

## Abstract

Cardiac anatomy and function vary considerably across the human population with important implications for clinical diagnosis and treatment planning. Consequently, many computer-based approaches have been developed to capture this variability for a wide range of applications, including explainable cardiac disease detection and prediction, dimensionality reduction, cardiac shape analysis, and the generation of virtual heart populations. In this work, we propose a variational mesh autoencoder (mesh VAE) as a novel geometric deep learning approach to model such population-wide variations in cardiac shapes. It embeds multi-scale graph convolutions and mesh pooling layers in a hierarchical VAE framework to enable direct processing of surface mesh representations of the cardiac anatomy in an efficient manner. The proposed mesh VAE achieves low reconstruction errors on a dataset of 3D cardiac meshes from over 1,000 patients with acute myocardial infarction, with mean surface distances between input and reconstructed meshes below the underlying image resolution. We also find that it outperforms a voxelgrid-based deep learning benchmark in terms of both mean surface distance and Hausdorff distance while requiring considerably less memory. Furthermore, we explore the quality and interpretability of the mesh VAE's latent space and showcase its ability to improve the prediction of major adverse cardiac events over a clinical benchmark. Finally, we investigate the method's ability to generate realistic virtual populations of cardiac anatomies and find good alignment between the synthesized and gold standard mesh populations in terms of multiple clinical metrics.

## 1. Introduction

Inter-subject variability in human cardiac anatomy and function plays a decisive role in the accurate diagnosis and treatment of many cardiovascular diseases, including myocardial infarction, heart failure, and reinfarction (1–3). Therefore, it is a key objective of computational models of the heart to be able to capture this variability across a population in order to obtain realistic representations of cardiac morphology and physiology. Such models not only enable a more accurate definition of shape normality within a given subpopulation but also improve the ability to detect abnormalities while retaining interpretability of the diagnosis (4, 5). Cardiac magnetic resonance imaging (MRI) is considered the gold standard imaging modality for the non-invasive assessment of cardiac anatomy and function in clinical practice (6). Accordingly, it has been extensively used as the basis to investigate anatomical shape variability in previous literature. While many works have focused on quantifying anatomical information based on the 2D slices of the underlying cine MRI acquisition (4, 7–10), this provides only an approximation of the heart's true 3D shape and therefore neglects more localized shape variability which is crucial for the detection and diagnosis of various cardiac diseases (2, 11, 12). Consequently, other works have conducted cardiac shape analysis directly on 3D representations of the heart which have either been reconstructed from 2D slices (13–18) or been acquired using a 3D MRI acquisition protocol (19). In order to study the anatomical variability in the obtained 3D heart shapes, principal component analysis (PCA) has been widely used in previous literature, as it allows to easily and quickly identify the most important modes of shape variation within the population (5). This low-dimensional representation of cardiac shape information can then be used for a variety of follow-up tasks, such as to investigate the association of shape and cardiovascular risk factors (20, 21), determine the probability of future major adverse cardiac events (MACE) (2), study the connection between shape and simulated cardiac function (22, 23), generate virtual population cohorts for *in silico* trials (24), or predict myocardial infarction (MI) (12). While the low-dimensional scores are obtained by PCA in an unsupervised manner, supervised methods, such as linear discriminant analysis (LDA) and information maximizing component analysis, have also been proposed to directly take into account information about the task-specific objective in the cardiac anatomy modeling (25).

More recently, deep learning approaches based predominantly on the variational autoencoder (VAE) (26) framework have been increasingly used to capture population-wide anatomical variability for a variety of tasks (5). Similar to PCA, the VAE allows for the representation of 3D shape information in a low-dimensional space with individual components corresponding to different aspects of inter-subject variability. However, in contrast to standard PCA or LDA approaches, VAEs are capable of modeling considerably more complex relations, primarily due to their deep learning-based architecture with a high number of trainable parameters and the presence of non-linear functions. The autoencoder structure with a low-dimensional latent space representation also allows for the straightforward integration with other common image-based tasks while maintaining a good degree of interpretability. Such tasks include the detection of coronary artery disease (27) and hypertrophic cardiomyopathy (28), image segmentation with shape priors (29–32), multi-task segmentation and regression (33), image-to-image synthesis (33), and survival prediction (34). However, the aforementioned approaches mostly rely on representing cardiac shapes as fixed-size 3D voxelgrids and use standard grid-based deep learning operations. This is not only inefficient in terms of memory and time requirements but also complicates effective feature learning when processing anatomical surface data. In order to overcome these issues, geometric deep learning techniques (35) have been introduced to enable accurate learning directly on non-Euclidean data, such as point clouds or graphs. This enables the anatomical surface information of the heart to be represented and processed in a highly efficient manner targeted to the data-type at hand and hence, has seen various applications in cardiac image analysis. For example, point cloud-based deep learning approaches have been proposed for the generation of virtual cardiac anatomies (36), classification of cardiac disease (37), modeling of 3D deformation of the heart (38), surface reconstruction of cardiac anatomy (13, 39), combined reconstruction and segmentation of the left ventricular (LV) wall (40), and the joint modeling of cardiac anatomy and electrocardiogram data (41, 42). Similarly, graph neural networks have been investigated for the simulation of cardiac mechanics (43), reconstruction of cardiac meshes (44), prediction of cardiac depolarization times (45), and the estimation of wall shear stress in 3D artery models (46).

Following these advancements, we propose in this work a variational mesh autoencoder (mesh VAE) as a novel approach to cardiac anatomy modeling. The mesh VAE is specifically designed to work directly on 3D mesh representations of the heart and thus overcomes the limitations of voxelgrid-based approaches. This enables the efficient processing of high-resolution 3D cardiac anatomy data and provides a more accurate modeling of 3D shape variability. The mesh VAE combines graph convolution and mesh sampling layers in a hierarchical setup to allow effective multi-scale feature learning of non-linear relationships. At the same time, the VAE framework ensures a high degree of interpretability with a disentangled, low-dimensional latent space. The architecture is also highly adaptable and can be used in combination with different imaging modalities, disease types, and application domains in a similar way as grid-based autoencoders. In summary, we make the following contributions in this work:

- We develop a novel variational mesh autoencoder for 3D cardiac anatomy modeling directly on 3D surface meshes.
- We successfully embed the mesh VAE into a multi-step cardiac anatomy modeling pipeline to enable clinical applicability.
- We evaluate the mesh VAE's ability to reconstruct high-resolution anatomy meshes on a multi-domain cine MRI dataset of myocardial infarction patients at both the end-diastolic (ED) and end-systolic (ES) phases of the cardiac cycle.
- We conduct a comparative analysis of the mesh VAE and a voxelgrid-based VAE benchmark in terms of both their reconstruction capabilities and technical specifications and demonstrate the advantages of the mesh VAE for the processing of anatomical surface data.
- We investigate the latent space of the mesh VAE as an efficient low-dimensional encoding of high-dimensional cardiac surface anatomy information in terms of its interpretability, disentanglement, association with generated output meshes, and accurate representation of inter-subject shape variability.
- We analyze the suitability of the mesh VAE for the generation of realistic virtual population cohorts of cardiac anatomy meshes.
- We explore the utility of the mesh VAE's latent space representations to capture pathology-specific shape biomarkers and predict MACE events in post-MI patients.
- We provide a pertinent literature review and a detailed discussion of the results including the proposed method's limitations and possible future use cases.

## 2. Materials and methods

In this section, we give an overview of the proposed cardiac shape modeling pipeline (Section 2.1), describe the dataset and preprocessing steps (Section 2.2) used for method development, and explain the architecture (Section 2.3), loss function (Section 2.4), and training procedure (Section 2.5) of the proposed mesh VAE network.

### 2.1. Overview

**Figure 1:**
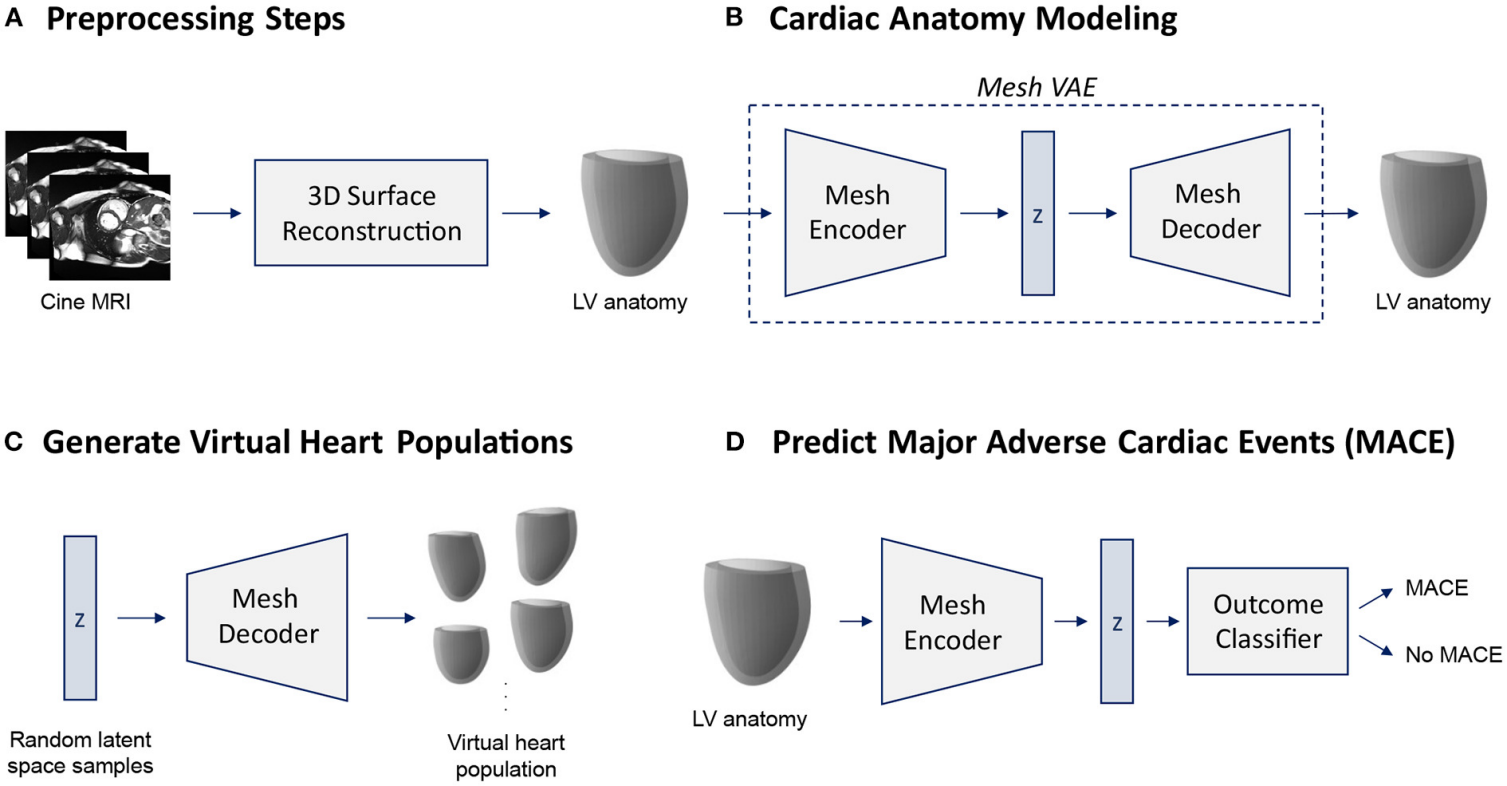
Overview of the proposed cardiac shape analysis pipeline **(A,B)** and the two possible applications investigated in this work **(C,D)**. First, we pass raw cine MRI acquisitions through a multi-step 3D surface reconstruction pipeline (Section 2.2) to reconstruct 3D surface meshes of the left ventricular (LV) anatomy as a preprocessing step **(A)**. It consists of an image segmentation step using two cascaded U-Nets followed by a personalized template mesh fitting step based on variational warping techniques (Section 2.2). Next, as the core part of the pipeline, we propose a variational mesh autoencoder with an interpretable latent space *z* to efficiently capture population-wide variability in 3D cardiac shapes in a single geometric deep learning model **(B)** (Section 2.3). The decoder of the pre-trained mesh VAE can then be used to generate virtual population cohorts of 3D heart meshes **(C)** (Section 3.5). The pre-trained encoder converts input meshes into low-dimensional latent space representations which can serve as inputs to a MACE outcome classifier **(D)** (Section 3.6).

In this work, we introduce a novel variational mesh autoencoder embedded into a multi-step pipeline to enable efficient non-linear 3D shape analysis of the human heart (Figure 1).

In the first part of our pipeline, we apply several preprocessing steps to prepare the raw images of our dataset for 3D shape modeling with the mesh VAE (Figure 1A). The input consists of the short-axis (SAX) slices of a standard cine MRI acquisition which we first segment using a two-step cascaded U-Net (47) approach (Section 2.2). Next, we fit a template mesh to the resulting SAX contours in a numerical optimization procedure in order to obtain 3D mesh representations of the cardiac anatomy (Section 2.2). We then use these 3D surface meshes to train and evaluate the proposed mesh VAE to capture cardiac shape variability across the population (Figure 1B). The mesh VAE constitutes the core part of the shape modeling pipeline with its architecture specifically tailored to process complex 3D surfaces of the cardiac anatomy (Section 2.3). This enables a variety of different clinical and research-related use cases. As two possible sample applications, we investigate both the generation of virtual populations of cardiac anatomy meshes using the mesh VAE's decoder (Figure 1C) and the binary prediction of MACE outcomes based on 3D cardiac shape information as encoded in the mesh VAE's latent space representations (Figure 1D). In the following sections, we describe each part of the pipeline in greater detail with a particular emphasis on the proposed mesh VAE.

### 2.2. Dataset and preprocessing steps

Our dataset consists of 1,021 post-MI patients for which cine MR images were acquired a median of 3 days after the infarction event in a multi-center study. It is based on both the TATORT-NSTEMI trials (Thrombus Aspiration in Thrombus Containing Culprit Lesions in Non-ST-Elevation Myocardial Infarction; NCT01612312) and the AIDA-STEMI trials (Abciximab Intracoronary vs. Intravenously Drug Application in ST-Elevation Myocardial Infarction; NCT00712101) and hence includes both Non-ST-Elevation Myocardial Infarction (NSTEMI) and ST-Elevation Myocardial Infarction (STEMI) patients (48–50). Electrocardiography-gated balanced steady-state free precession sequences were used for all acquisitions. The pixel resolution varied across the acquired images with a mean value of 1.36 mm (range: [1.16, 2.08] mm) and standard deviation (SD) of 0.21 mm. Each patient was followed up for 12 months post-MI with MACE (reinfarction, new congestive heart failure, or all-cause death) defined as the clinical endpoint. Overall, 74 patients experienced a MACE outcome. Further details regarding the study population and image acquisition can be found in (2, 48–50).

We first apply a multi-step preprocessing pipeline to reconstruct 3D surface mesh representations of the left ventricular anatomy from the raw cine MRI acquisitions. The first step of this pipeline consists of the segmentation of left ventricular (LV) myocardium on the cine cardiac MRI using two cascaded U-Nets with enhanced preprocessing (51, 52). The first U-Net locates the LV to crop and orient the images accordingly, while the second U-Net performs the fine segmentation. This architecture addresses both canonical orientation for regional metrics quantification and label imbalance for segmentation performance improvement. Next, two personalized 3D LV meshes at the ED and ES phases are built from the segmentation contours for each patient. The reconstruction of these 3D meshes uses a solution based on smooth cubic Hermite interpolation, where, in brief, an idealized LV template mesh is fitted to the 3D myocardium segmentation mask by combining image registration and mesh projection techniques (17, 53, 54). The Hermite template mesh is an idealized LV (truncated ellipsoid of 6 longitudinal × 12 circumferential × 1 radial elements). Since the same template is used for all the patients, homologous points are directly obtained. Further details on the pipeline can be found in (2). The resulting 3D surface meshes of the left ventricular anatomy are then used as inputs for training and evaluating the mesh VAE. We split the mesh dataset into 70% training, 5% validation, and 25% test datasets while maintaining the same class imbalance between MACE and no MACE cases in each subset. Finally, we apply standardization (i.e., subtracting the mean and dividing by the SD) to each mesh before inputting it into the network.

### 2.3. Variational mesh autoencoder architecture

**Figure 2:**
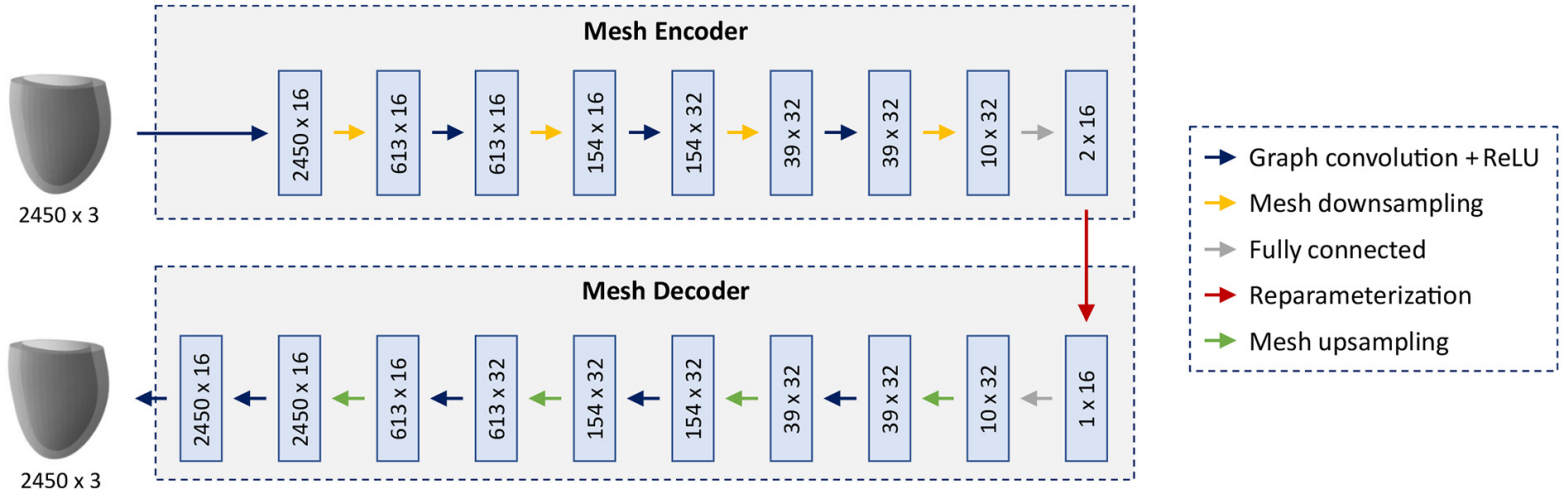
Architecture of the mesh VAE. The input and output are cardiac anatomy models represented as 3D triangular meshes. All meshes in the dataset share the same connectivity and consist of 2,450 vertices with associated x,y,z coordinates. Multi-scale feature learning directly on mesh data is enabled by alternating graph convolution and sampling operations which are arranged in a hierarchical setup. The mesh encoder and decoder, connected by a 16-dimensional latent space, follow a symmetric design with the same number of levels and the same mesh resolution per level.

As the core part of our shape modeling pipeline, we propose a novel mesh VAE architecture, specifically designed based on recent advances in mesh-based deep learning (55, 56) to efficiently process triangular mesh data of the 3D cardiac anatomy (Figure 2).

The overall architecture consists of an encoder and a decoder connected by an interpretable 16-dimensional latent space with the ability to capture high-dimensional cardiac shape information in a low-dimensional representation. The building blocks of the network follow recent advances in mesh-based deep learning to enable effective learning of non-linear relationships directly on triangular mesh data. The main feature extraction is accomplished by graph convolution blocks which are composed of spectral graph convolutional layers (56) followed by rectified linear unit (ReLU) activation functions. Multiple mesh downsampling operations (55) are positioned between successive graph convolution blocks along the encoder to allow for stepwise decreases in mesh resolution and multi-scale hierarchical learning. The decoder follows a symmetric design to the encoder with mesh upsampling operations interspersed between graph convolution blocks and the same mesh resolutions, number of levels and feature maps as the encoder. This enables the decoder to reconstruct high-resolution anatomical meshes from the latent space in a gradual multi-scale process akin to the stepwise encoding operation of the encoder. Fully connected layers are introduced before and after the latent space to connect the tensors representing downsampled mesh information and the latent space in an effective way. All spectral graph convolutions use the Chebyshev polynomial approximation (56, 57) of order 5 for efficient calculation. The mesh sampling operation uses quadric error minimization to identify the vertices that are removed in the downsampling step, then saves their location in barycentric coordinates, before using these coordinate values to reinsert them in the upsampling step (55). All input anatomies are represented as 3D triangular surface meshes with 2,450 vertices and identical vertex connectivity across the dataset.

### 2.4. Loss function

The loss function of the proposed mesh VAE is based on the β-VAE framework (58) and consists of the sum of a reconstruction loss term and a Kullback-Leiber (KL) divergence term, weighted by a parameter β.


(1) $$\begin{array}{l} L_{total}=L_{reconstruction}+\beta *L_{KL}. \end{array}$$

The weighting parameter β is used to control the importance of each of the two loss terms during training. Similar to previous approaches for cardiac shape analysis using point cloud deep learning (36), we follow a monotonic annealing schedule (59) for β and set it to small values (starting at 0.0001) at the beginning of training before gradually increasing it until 0.001 at the end of training. This allows the network to put more emphasis on the accurate mesh reconstruction task first and then step-by-step focus more on also achieving a high latent space quality. The Kullback-Leibler divergence term in the total loss function used in this work is defined as follows:


(2) $$L_{KL}=D_{KL}\left[Q(z\text{|}X)\text{‖}P(z)\right].$$

Here, *X* refers to the input mesh, *z* to the latent space of the mesh VAE, and *Q*(*z*|*X*) to the posterior distribution of the VAE's latent space. *P*(*z*) is the prior distribution of the VAE's latent space for which we choose a multivariate standard Gaussian distribution in this work. This helps the VAE to achieve a smooth and disentangled latent space, thus improving the representation of cardiac shape variability.

We select the mean squared error (MSE) between the coordinate values of the corresponding vertices *n* in the input mesh *x* and the reconstructed mesh *y* as our reconstruction loss term. This encourages the VAE to put more emphasis on larger vertex distances between input and ground truth meshes which facilitates the task of capturing the full extent of cardiac shape variability across the population.


(3) $$\begin{array}{l} L_{reconstruction}=\frac{1}{N}\overset{N}{\underset{n=1}{\sum }}{\left(x_{n}-y_{n}\right)}^{2} \end{array}$$

### 2.5. Network training and implementation

We train the mesh VAE for 250 epochs with a learning rate of 0.001 and a batch size of 8 using the Adam optimizer (60) on a CPU. The reparameterization trick (26) is applied during training. All general deep learning code in this work was based on the PyTorch framework (61), while the PyTorch Geometric library (62) was used for graph-specific deep learning operations. The machine learning classifiers for the experiments in Section 3.6 were implemented using the scikit-learn library (63).

## 3. Experiments and results

We evaluate the proposed mesh VAE in a variety of different settings using various evaluation metrics (Section 3.1) to showcase its versatility and demonstrate its usefulness in multiple applications related to cardiac shape analysis. These include an assessment of its ability to accurately reconstruct 3D cardiac mesh inputs (Section 3.2), a comparative analysis with a voxelgrid-based deep learning benchmark (Sections 3.2, 3.3), and an investigation of its latent space quality (Section 3.4). In addition, we evaluate its ability to generate realistic virtual population cohorts of cardiac anatomies (Section 3.5) and the utility of its latent space to predict MACE events (Section 3.6) as two possible sample applications of the mesh VAE.

### 3.1. Evaluation metrics

We utilize multiple metrics in this work to enable a thorough evaluation of the mesh VAE in a variety of settings and tasks.

We select the mean surface distance (MSD) (Equation 4) between two triangular meshes *X* and *Y* as our first metric to quantify the averaged difference between two anatomical surfaces. This allows the assessment of the general alignment between our method's predictions and the corresponding gold standard.


(4) $$MSD(X,Y)=\frac{1}{2}\left(\frac{1}{\left|X\right|}\sum_{x\in X}d(x,Y)+\frac{1}{\left|Y\right|}\sum_{y\in Y}d(y,X)\right)$$

In addition to the average distance between two meshes, we also want to obtain the maximum difference between the two. This allows us to see whether larger deviations are present in smaller regions on the mesh surfaces which is important for a more localized cardiac shape analysis. We choose the Hausdorff distance (HD) between input meshes *X* and *Y* for this purpose.


(5) $$HD(X,Y)=\max\{\underset{x\in X}{\sup}\underset{y\in Y}{\inf}d(x,y),\underset{y\in Y}{\sup}\underset{x\in X}{\inf}d(x,y)\}$$

While MSD and HD provide a geometric quantification of mesh alignment, we also aim to provide an assessment in terms of commonly used clinical metrics to facilitate the proposed method's application in clinical practice. To this end, we choose the LV endocardial volume and the LV myocardial mass to validate the anatomical realism of the virtual meshes generated by our method across a given population. Here, we define the LV mass as the difference between LV epicardial and LV endocardial volumes multiplied by a constant (ρ = 1.05*g*/*mL*) to represent the average density of the myocardial tissue.

An advantage of any variational autoencoder architecture is the existence of a latent space that aims to provide an accurate low-dimensional and disentangled representation of the high-dimensional population distribution. In order to improve interpretability and performance on multiple follow-up tasks, each dimension of the latent space ideally encodes a different aspect of the inter-subject anatomical variability. To this end, we quantify the contribution of each latent space dimension *u* using the activity metric (64).


(6) $$\begin{array}{l} Activity_{u}=Cov_{X}\left({\text{𝔼}}_{u\sim q\left(u|X\right)}\left[u\right]\right) \end{array}$$

Here, *X* refers to the input meshes, 𝔼 to the expected value, *Cov* to the covariance, and *q* to the posterior probability distribution of the latent space component *u*. Intuitively, a higher activity score indicates that a larger amount of population-wide shape variability is captured by the given latent space dimension.

Finally, in order to assess the utility of the mesh VAE's latent space representation for the binary MACE prediction task, we select the area under the receiver operating characteristic (AUROC) curve as our metric due to the class imbalance in our dataset.

### 3.2. Mesh reconstruction

**Figure 3:**
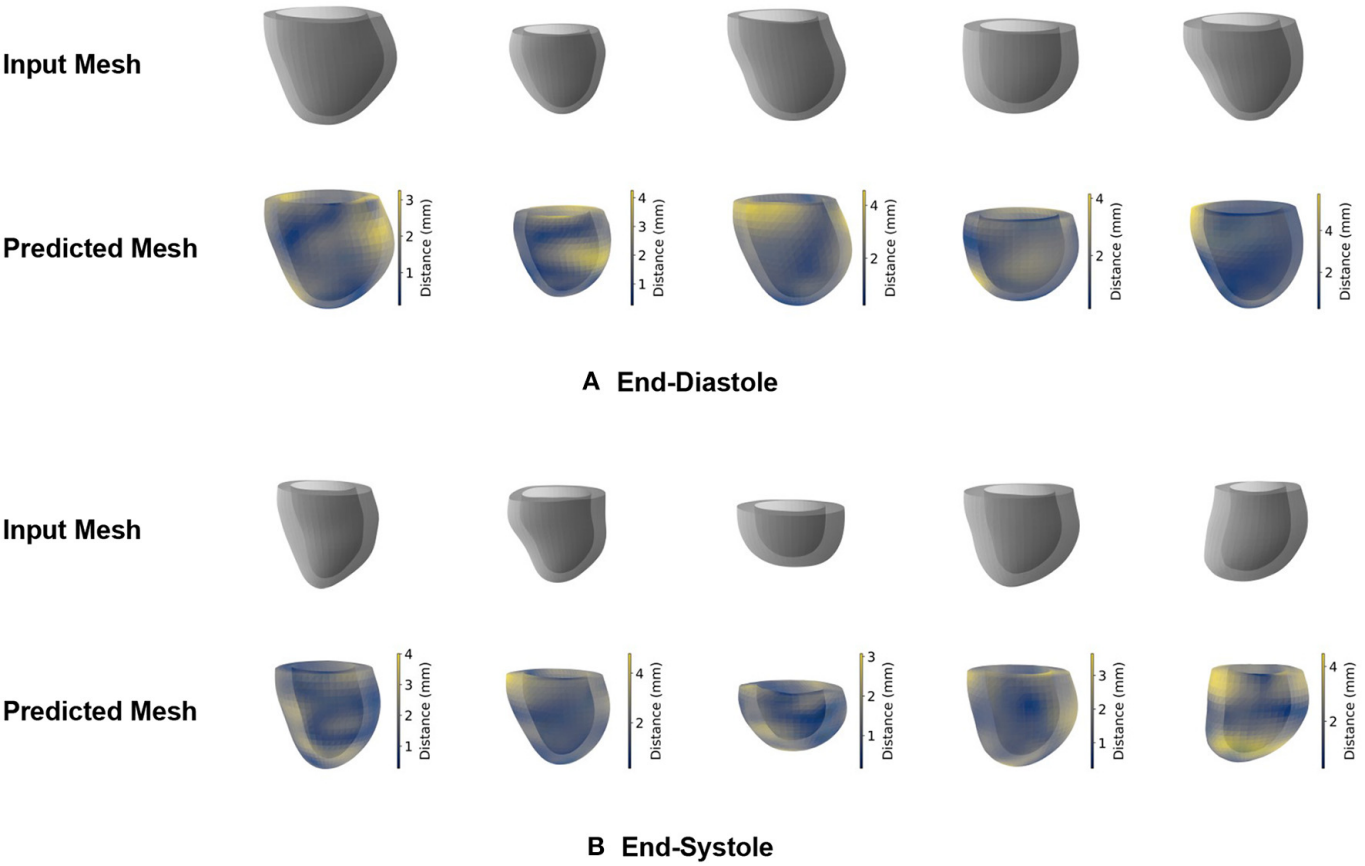
Qualitative reconstruction results of the mesh VAE for five sample cases. Results are presented separately for ED **(A)** and ES **(B)** phases. Predicted meshes are color-coded based on the vertex-wise distances to their corresponding input meshes.

In our first experiments, we want to assess whether the proposed mesh VAE is capable of accurately encoding and reconstructing the complex high-dimensional anatomical meshes at both the ED and ES phases of the cardiac cycle. To this end, we first train separate mesh VAE models for each of the two cardiac phases and evaluate their reconstruction performance qualitatively by comparing the output meshes with the corresponding input meshes of the unseen test dataset. The obtained results for five sample cases are depicted for both ED and ES phases in Figures 3A,B, respectively.

We observe that the shapes of the input and reconstructed meshes closely resemble each other on both a local and global level and for both the ED and ES phases. The relationship between epicardial and endocardial surfaces remains consistent between input and predicted meshes, while the most noticeable differences appear in regions with remaining slice misalignment artifacts and at the base of the left ventricular anatomy. We also notice a slight smoothing effect of the mesh VAE outputs compared to the respective input meshes, especially in localized regions affected by surface reconstruction artifacts.

In order to quantify the mesh VAE's encoding and reconstruction ability, we calculate both the mean surface distance (Equation 4) and the Hausdorff distance (Equation 5) between the predicted meshes and gold standard meshes of the test dataset (Table 1). This enables an assessment of both the average and worst-case performance of how accurately the mesh VAE can process unseen 3D shape information. As a benchmark method for comparative validation, we choose a 3D VAE which is designed for 3D voxelgrid data and has previously been used to process 3D cardiac surface information (28, 65). We train and evaluate the 3D VAE on the same training and test datasets as our mesh VAE and report the results in Table 1. In order to apply the 3D VAE to our mesh dataset, we first voxelize each 3D triangular mesh and then place it in the center of a 128 × 128 × 128 voxelgrid with a voxel size of 1.5 × 1.5 × 1.5 mm. The resulting voxelgrids serve as the input and gold standard data for the 3D VAE. The architecture, loss function, and training procedure of the 3D VAE are chosen to be as close as possible to the mesh VAE's design in order to enable a fair comparison. The graph convolutions and mesh pooling layers are replaced by standard convolution and max pooling operations, respectively.

**Table 1:** Reconstruction results of the mesh VAE and the 3D VAE on the test dataset.

**Phase**	**Method**	**Hausdorff distance (mm)**	**Surface distance (mm)**
ED	3D VAE	6.43 (±2.57)	1.26 (±0.48)
ES	3D VAE	6.64 (±2.81)	1.53 (±0.66)

Values represent mean (± standard deviation) in all cases.

We find that the mesh VAE obtains mean surface distance values considerably below the pixel resolution of the underlying image acquisition (1.36 mm) for both ED and ES phases. It also achieves significantly lower distance scores than the 3D VAE for both HD and MSD metrics and for both ED and ES phases. For both evaluated methods and phases, the Hausdorff distance values are substantially larger than the MSD scores indicating that certain small localized regions exhibit larger differences between reconstructed and gold standard meshes than the global average.

### 3.3. Technical comparison

In addition to assessing the mesh VAE in terms of its reconstruction performance, we also evaluate its memory footprint in comparison to the 3D VAE. To this end, we calculate both the size of each data instance used as an input to the respective networks and the number of trainable network parameters in each approach (Table 2).

**Table 2:** Technical comparison of the mesh VAE and the 3D VAE.

**Method**	**Data type**	**Data instance size**	**Network parameters**
3D VAE	Voxelgrid	~2.1 × 10^6^ (128 × 128 × 128)	~1.1 × 10^6^
Mesh VAE	Mesh	~7.4 × 10^3^ (2450 × 3)^*^	~4.4 × 10^4^

* Vertex connectivity is the same for each mesh in the dataset.

In terms of both metrics, the mesh VAE shows considerably better scores than the 3D VAE. It requires only about 25 times fewer trainable network parameters and processes approximately 285 times smaller input data while still representing the underlying cardiac anatomy with higher fidelity. This also allows us to train the mesh VAE on a standard CPU as opposed to the GPU required for the 3D VAE. While this makes a direct comparison of the run time difficult, we would expect it to lead to a considerably faster execution of the Mesh VAE.

### 3.4. Latent space analysis

After having shown the mesh VAE's ability to accurately model 3D cardiac shapes with high efficiency, we want to further investigate its latent space as a key architectural component to successfully represent inter-subject anatomy changes. As indicated by the two terms in the VAE's loss function, the objective of the latent space is similarly two-fold. On the one hand, it aims to provide a suitable low-dimensional encoding of high-dimensional input meshes that allows for accurate reconstruction. On the other hand, it is also tasked to represent important aspects of population-wide shape variability in a disentangled and interpretable way by approximating a multivariate Gaussian distribution. While the experiments in Section 3.2 show the adequacy of the mesh VAE's latent space for the reconstruction task, we want to focus on its role in modeling variability in this section.

**Figure 4:**
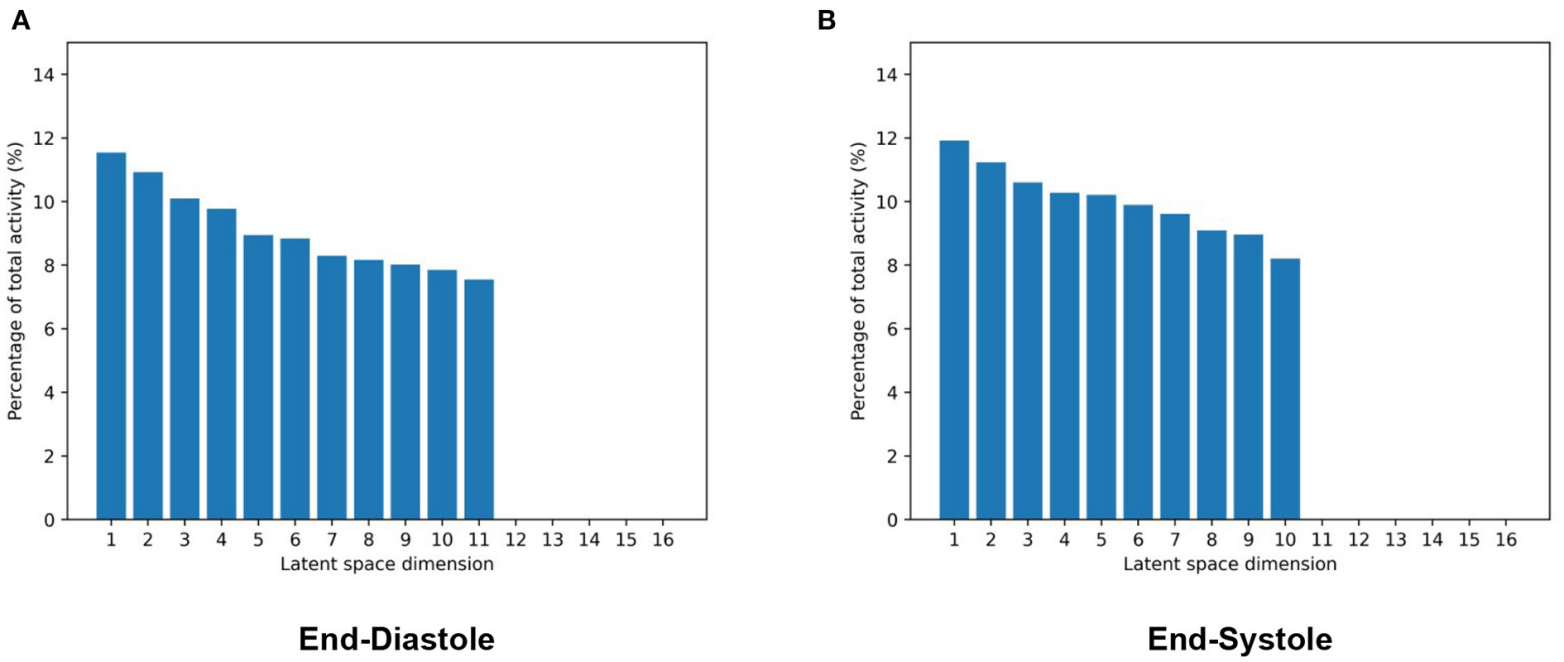
Activity values of each latent space dimension as a percentage of total activity in mesh VAEs trained separately on ED **(A)** and ES **(B)** data. Latent space dimensions are presented in decreasing order of their activity percentage values.

To this end, we first pass all meshes of our dataset through the pretrained encoder of the mesh VAE to obtain the latent space representation of each case. We then use these representations to calculate the activity of each latent space component. Intuitively, the activity scores give an indication as to how much of the overall shape variability across the population is captured by a given latent space dimension. We follow these steps separately for the mesh VAEs trained on ED and ES data, respectively, and report the results in Figure 4. Hereby, the activity of each latent space dimension is presented as a percentage of the total activity, and latent space dimension are arranged in decreasing order of their respective activity percentages.

We observe that while the majority of the 16 latent space components capture more than 8% of the overall population variability each, a few components model close to 0%. Among the significantly contributing components, differences between the most and least active dimensions are relatively small at approximately 3%. Results are mostly consistent between the ED and ES phases with the ED phase showing one additional significantly contributing component and consequently slightly smaller activity scores for each of them.

**Figure 5:**
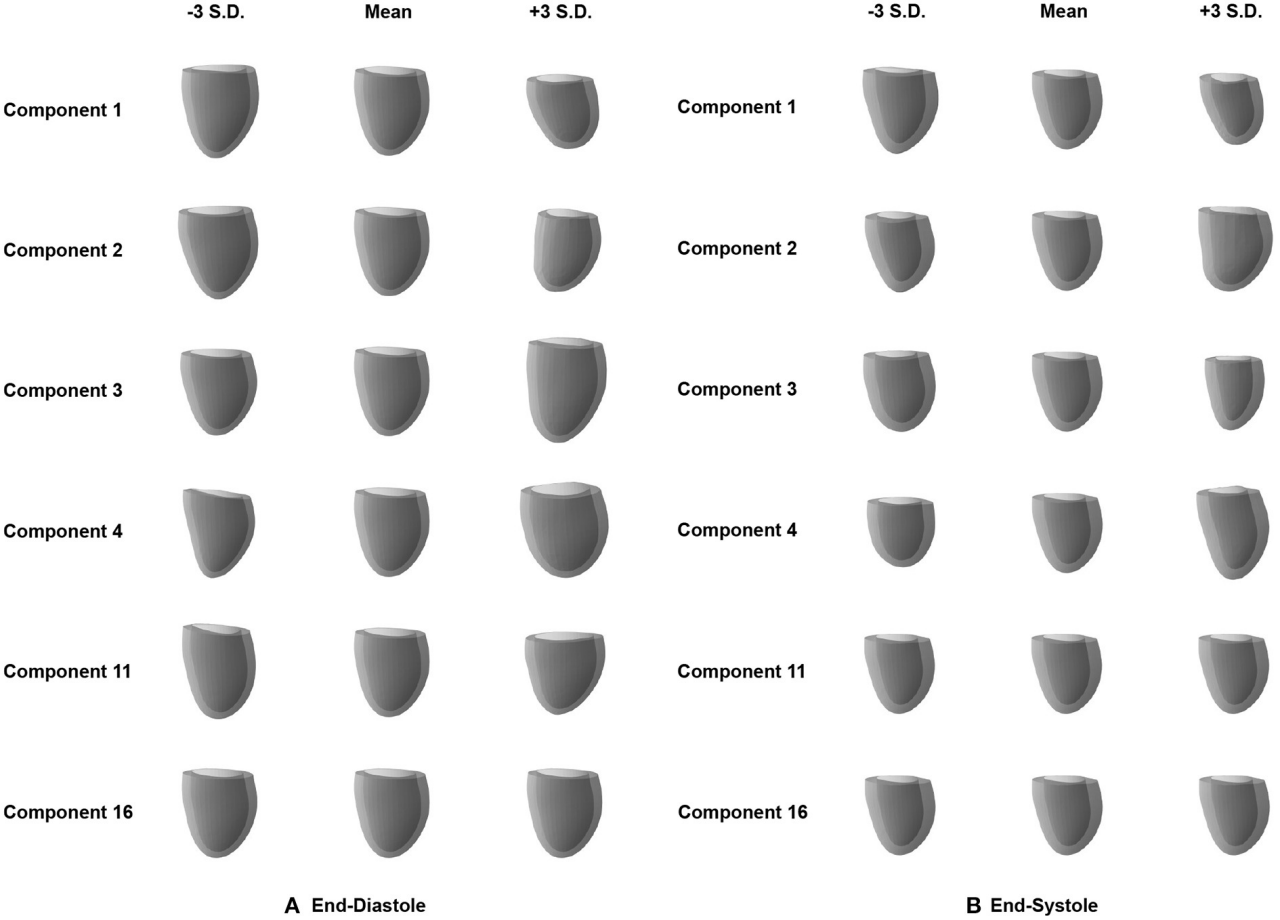
Effect of changes in the individual latent space components (rows) on the 3D mesh shapes reconstructed by the decoder of the mesh VAE for ED **(A)** and ES **(B)** phases. Results are shown for the four most active latent space components (1–4), the least active one (16), and the one with the largest activity difference between ED and ES phases (11).

After quantifying the variability in the low-dimensional latent space, we want to investigate the effect of changes in the individual latent space components on 3D shape variability. This allows us to identify if different latent space dimensions are responsible for modeling different aspects of the 3D anatomical variability. To this end, we first determine the mean latent space encoding across the population and then vary individual latent space components while keeping the other components fixed at the population mean value. We next pass these latent space representations through the decoder of the pre-trained mesh VAE in order to visualize the effect of the change in the particular latent space component on the 3D anatomy. Each individual latent space component is varied by 3 standard deviations from its mean value in both the positive and negative directions to analyze shape changes in both sides of the unimodal probability distribution. Based on our findings in Figure 4, we depict the resulting meshes corresponding to changes in the four most active latent space components, the least active one, and the one with the largest activity difference between ED and ES phases in Figure 5.

We observe that variations in each of the four most active latent space components result in gradual and easily identifiable changes in 3D anatomical shapes for both the ED and ES phases. These include changes in overall heart size, the pointedness of the apex, the basal plane tilt, and the longitudinal curvature and elongation of the ventricle. Variations along the least active latent space component do not cause any easily noticeable changes to the overall shape in either the ED or ES phase. We also find clear 3D shape changes when varying the last component with significant activity scores for the ED phase (component 11), while the same component for ES phase represents the first component with low activity scores and does not produce any easily visible 3D shape changes.

### 3.5. Generation of virtual cardiac mesh populations

**Figure 6:**
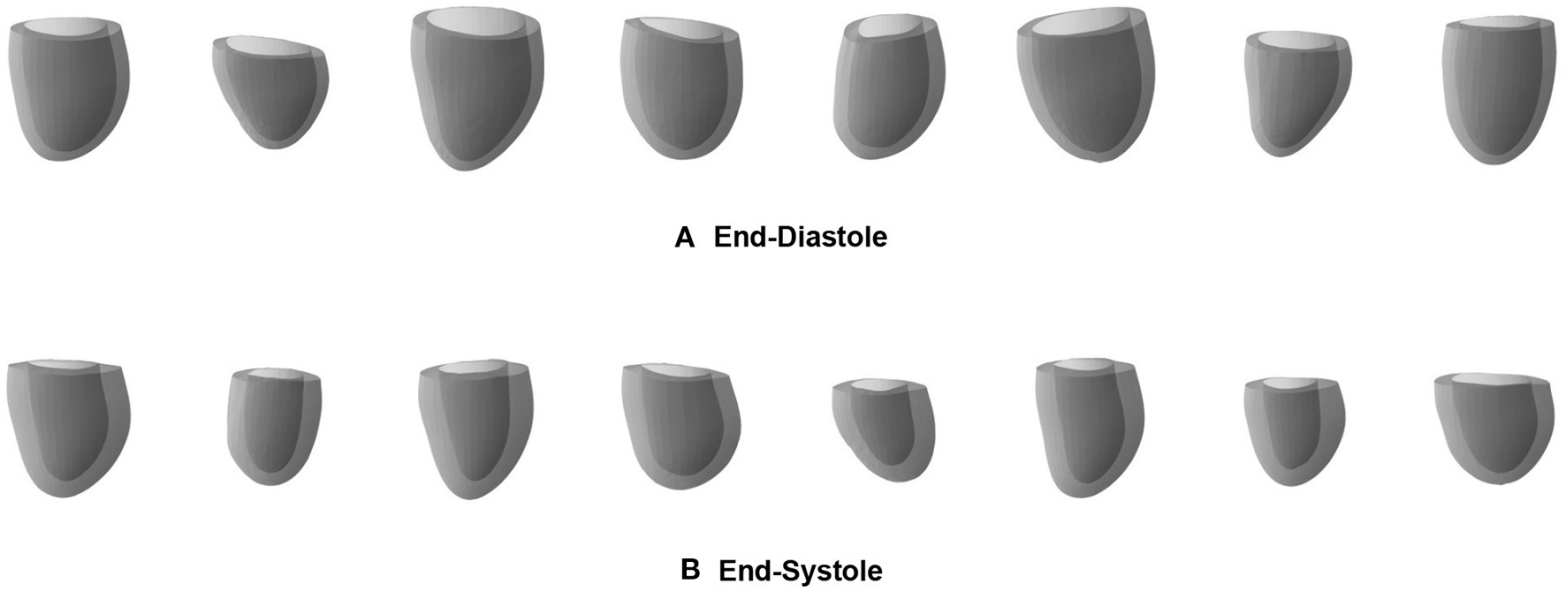
Sample meshes generated by separate pretrained ED **(A)** and ES **(B)** mesh VAEs.

As a first possible sample application of our mesh VAE, we next want to analyze whether it is able to generate new as well as realistic 3D cardiac meshes altogether. Such virtual population cohorts have a variety of use cases, such as data augmentation for disease classification or electrophysiological computer simulations as part of *in silico* trials. Hereby, the synthesized meshes should be as indistinguishable as possible from the real ones on both an individual and a population level. In order to evaluate the mesh VAE's performance in this task, we draw random samples from the multivariate latent space distribution and pass each of them through the trained decoder of the VAE to obtain the corresponding virtual output meshes. We perform this procedure separately for the mesh VAE decoder trained on ED and ES and depict 8 sample results for each phase in Figure 6.

We observe that the generated meshes exhibit a degree of shape variation close to that of the true population for both the ED and ES phases, while still maintaining realistic anatomical shapes on an individual level. Typical shape changes regarding for example the overall heart size, mid-cavity diameter, or basal plane tilt, are successfully represented in the virtual population. In addition, the heart meshes at ES phase generally show a thicker myocardium and a smaller overall volume than the ED population, which is again reflective of the true population. Since we use separate networks for the ED and ES phases, no per-case correspondence between individual generated meshes of the two phases is enforced.

In order to quantify the realism of the generated heart population, we first randomly sample 1,000 latent space vectors and pass them through the mesh VAE's decoder to obtain a large virtual population of 3D cardiac meshes. We then calculate the widely used clinical metrics LV volume and LV mass for each mesh in the generated population and report the resulting population mean and standard deviation values in Table 3. For a comparative analysis, we also provide the same scores for the meshes in the unseen test dataset, which we assume as our gold standard in this work. We apply this procedure for both ED and ES phases using the respective pre-trained networks and report the results separately for each phase in Table 3.

**Table 3:** Clinical metrics of generated and gold standard mesh populations.

**Phase**	**Clinical metric**	**Gold standard**	**Mesh VAE**
ED	LV volume (ml)	156 (±42)	152 (±40)
ES	LV volume (ml)	81 (±32)	79 (±28)

Values represent mean (± standard deviation) in all cases.

We find similar mean and standard deviation values between the synthesized mesh population and the gold standard mesh population for both evaluation metrics and cardiac phases. The average difference in population means across all scores is 2.5%, with slightly larger deviations for the ED phase compared to the ES phase.

### 3.6. MACE prediction

**Figure 7:**
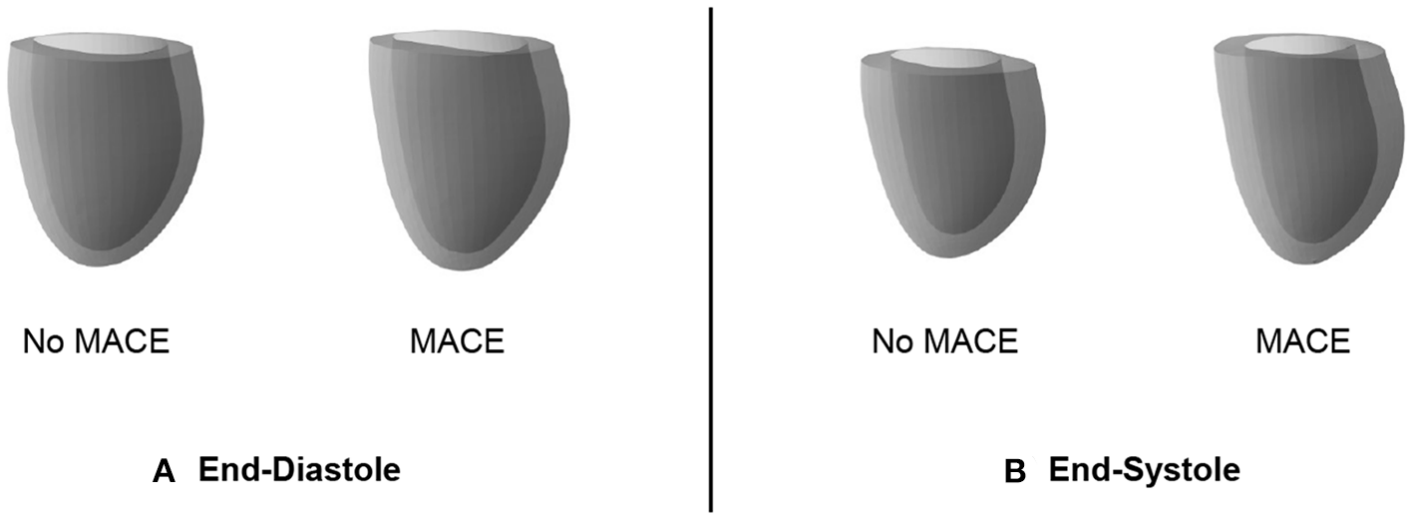
Cardiac anatomy meshes reconstructed by decoders of pre-trained mesh VAEs from averaged latent space representations of patients with and without subsequent MACE. Results are shown for ED **(A)** and ES **(B)** data.

In addition to its utility for generating virtual mesh populations, we also want to investigate whether the mesh VAE can capture pathology-specific information that is useful for cardiac disease detection and diagnosis. In this work, we focus on MACE as a possible sample outcome and want to first study whether there exist differences in the mesh VAE's latent space representations of post-MI subjects with and without an associated MACE. We therefore pass all MACE cases through the encoder of the pre-trained mesh VAE, average the resulting latent space representations, and then feed the resulting mean vector through the pre-trained decoder to obtain the average mesh representation of all MACE cases in the population. We repeat the same process for all cases without MACE and depict the obtained averaged meshes in Figure 7A for ED data and in Figure 7B for ES data.

We observe that the differences between averaged MACE and no MACE anatomies are small for ED data, but easily visible for ES data. This indicates that ED shape alone is considerably less predictive of incident MACE outcomes than ES shape, which is in line with clinical guidelines and previous research work (2).

Since the observed differences in 3D ES shapes between averaged MACE and no MACE cases were obtained using the same pre-trained decoder for mesh reconstruction, they should be caused by corresponding differences in the mesh VAE's latent space representations. As a next step, we want to investigate whether these low dimensional representations of 3D cardiac anatomies are not only suitable to represent subpopulation-specific average shapes, but also to predict future MACE outcomes for individual patients. To this end, we employ a logistic regression classifier to predict the binary outcome MACE vs. no MACE based on per-patient latent space encodings obtained from the mesh VAE encoder. As a clinical benchmark, we select the ES volume as a widely used metric in clinical practice and use it as the input to the another logistic regression model with the same settings. We choose the AUROC as a comparative metric for binary prediction performance due to the high class imbalance between MACE and no MACE cases in the dataset. In order to maintain the same class imbalance in the respective train and test sets and to improve the robustness of our analysis, we conduct stratified 10-fold cross validation experiments with both classifiers and report the averaged results in Table 4.

**Table 4:** Results of binary MACE classification.

**Metric**	**ES volume**	**Mesh VAE**
AUROC	0.627 (±0.042)	0.671 (±0.038)

Values represent mean (± standard deviation).

We find that the mesh VAE's latent space representations achieve an about 7% higher AUROC score than the ES volume values for the task of binary MACE prediction. We note, however, that the primary objective of this experiment is only to showcase the utility of the mesh VAE for a possible clinical application and not to present a method optimized for MACE prediction specifically, which we leave for future work.

## 4. Discussion

In this work, we have presented a novel geometric deep learning approach specifically designed for cardiac mesh processing as part of a multi-step cardiac shape analysis pipeline and demonstrated its versatility in multiple applications.

### 4.1. Mesh reconstruction

In our experiments, we find that the mesh VAE is able to accurately encode and decode complex 3D cardiac anatomy shapes with high degrees of realism, by attaining average surface distances between predicted and ground truth anatomies in the test dataset smaller than the underlying image resolution. This demonstrates that it is not only capable of capturing anatomical surface information in individual cases, but also correctly represents population-wide variability in cardiac shapes. These results are consistent across both the ED and ES mesh datasets and show that the mesh VAE is suitable for processing cardiac anatomy data at various phases of the cardiac cycle, albeit with separate networks for each phase. As these represent the two extreme ends of the cardiac cycle, we hypothesize that an application to intermediate frames is equally feasible. We observe this good alignment between predicted and input meshes not only on a global but also on a local surface level. This indicates that inter-subject shape variation is also successfully captured on a smaller, more localized scale which promises to aid in the discovery of new image-based biomarkers of cardiac abnormalities that go beyond the purely volume-based metrics widely used in current clinical practice. The largest localized prediction errors occur at regions with remaining slice misalignment artifacts and near the base of the left ventricle. We believe this to be at least partially a consequence of the limitations of the 3D surface reconstruction step (Section 4.6) rather than an issue of the mesh VAE itself. In fact, the observed slight smoothing effect of the mesh VAE typically occurs in localized regions that are still affected by reconstruction artifacts. This hints at potentially favorable effects of this small smoothing behavior, since its implicit slight misalignment corrections often result in more anatomically plausible 3D meshes without significant loss of true localized shape details. Multiple ways that would likely reduce the smoothing effect can be easily integrated into our mesh VAE framework, such as increasing the weight value of the reconstruction loss term during training or using a reconstruction loss that puts a disproportionately higher penalty on larger vertex-wise reconstruction errors. However, such measures could also lead to other unwanted effects, such as a reduced quality of the latent space distribution or an increased number of unnatural local deformations in the output meshes that mimic errors in the original 3D surface reconstruction process. In general, we note that the anatomically accurate reconstruction results have been achieved on a challenging dataset of pathological subjects acquired from multiple studies, in contrast to more homogeneous datasets of healthy subjects, such as the UK Biobank study (66). This further demonstrates the robustness of our mesh VAE.

### 4.2. Latent space quality

The ability of the mesh VAE to successfully model 3D cardiac shape variability across the population is further corroborated by the analysis of its latent space. We find that variations in latent space components are associated with realistic changes in reconstructed 3D shapes and that individual components are responsible for encoding different aspects of the population-wide shape variability. Examples of such easily visible effects include changes to the overall heart size, mid-ventricular diameter, and basal plane tilt, which are all similar to previous findings in cardiac shape modeling (19). This high degree of disentanglement enables an improved understanding of the key components of cardiac shape variations and higher levels of interpretability for the multiple clinical applications of the mesh VAE. When comparing the contribution of individual latent space components to overall shape variability, we find that some components are responsible for a larger percentage of the total variation than others. This is similar to the results of other widely used shape analysis techniques, such as PCA, where different principal components account for different proportions of the overall variance. Contrary to PCA, however, we find that the differences in activity percentage scores decrease only very slowly for the majority of components before a sharp drop after the 11th and 10th most contributing components for ED and ES data, respectively. All following components play almost no part in explaining the population variance. This is in contrast to PCA where the percentage of explained variance by each component typically decreases sharply for the first few most contributing components with very little change between less contributing components (2, 19–21). We hypothesize that this is due to the non-linearities in the mesh VAE's architecture which enable the modeling of richer and more condensed relationships between high-dimensional input data and low-dimensional latent space representations compared to purely linear approaches, such as PCA. This results in a different way of encoding shape variability with more equal activity scores for each contributing component. When varying along the latent space components with close to zero activity and observing the effect on the reconstructed 3D anatomies, we indeed find very little change, especially on a global level (Figure 4). However, similar to PCA, such components might still encode meaningful information about smaller, more localized shape variations. This induces a certain amount of risk when removing seemingly non-contributing components post-training, as otherwise important variability might be inadvertently removed. When experimenting with different latent space sizes in our mesh VAE, we find that differences in reconstruction accuracy are minimal between larger and smaller latent space dimensionalities. We also observe that 5–50% of the latent space dimensions have minor contributions to the overall variance, regardless of the choice of latent space size. Hence, we reason that the more condensed encoding of the same amount of shape information is a property of the overall mesh VAE architecture itself rather than solely a consequence of the latent space size. This also shows that changing the latent space size has little effect on removing potentially redundant latent space dimensions as the network adjusts its encoding accordingly. Furthermore, we also notice that training the same network with the same parameter settings can result in varying numbers of significantly contributing latent space dimensions. This indicates that the various sources of randomness involved in training deep learning networks (e.g., trainable parameter initialization, order of cases seen during training) affect the way the mesh VAE encodes shape information in the latent space. As such, we conclude that our choice of 16 as the latent space size reflects a reasonable trade-off between having too many modes and a representation that is too condensed, both of which would negatively affect interpretability. This also means that our choice is not a fixed optimal value but rather that it should be chosen with the particular dataset and downstream application in mind. When comparing the results for ED and ES meshes, we find very minor differences with only one additional contributing latent space component for ED and similar levels of disentanglement. We therefore conclude that the mesh VAE can successfully capture 3D shape variability at different phases of the cardiac cycle.

### 4.3. Generation of virtual cardiac mesh populations

In addition to accurately capturing 3D anatomical patterns of existing subjects, we also find that the mesh VAE is capable of generating realistic virtual populations of 3D heart meshes. Hereby, we observe a high degree of realism in both the individually generated meshes and in the amount of variability present in the overall virtual population, which closely mimics the true underlying population. We have shown this both qualitatively and quantitatively by calculating commonly used clinical metrics on a population level. We also note that we find not only a good alignment between virtual generated population and gold standard population in terms of their mean values but also in terms of their standard deviation scores, indicating that the variability across the population is well-captured in the virtual population. The mesh VAE achieves these positive results for both ED and ES phases which shows its versatility to different datasets and suggests the possibility of a feasible extension to other phases of the cardiac cycle. For example, the generated ES anatomies typically exhibit a thicker myocardium and an overall smaller size than their ED counterparts which is reflective of real cardiac morphology. The high degree of realism in the generated meshes is also visible on both a global and local level. This is particularly important for virtual population cohorts used in computer simulations of cardiac electrophysiology which model conduction patterns granularly for each face in a mesh. We also note that all generated virtual meshes retain the same vertex connectivity as a result of the chosen network architecture which is another beneficial property for many follow-up tasks. In our experiments, we find that sampling from a latent space distribution based on encoder predictions of the training dataset leads to better mesh generation results than sampling from a multivariate standard Gaussian distribution. We attribute this to the trade-off between the reconstruction and latent space terms in the loss function which cause the latent space to only approximate the idealized prior distribution in order to retain a high reconstruction accuracy.

### 4.4. MACE prediction

As a compact low-dimensional representation of high-dimensional cardiac anatomies, the latent space should also be able to capture subpopulation-specific differences based solely on information in the input shapes. In our experiments, we observe such differences for the MACE vs. no MACE subpopulations in the ES data and to a lesser extent in the ED data. In both cases, we hypothesize this to be a reflection of the information contained in the 3D shapes instead of a potential inadequacy of the latent space itself. This is corroborated by findings in previous work and clinical practice where metrics based on ES heart shapes are considerably more predictive than corresponding ED-based scores (2). We then show how these latent space differences for ES data can be successfully used to predict MACE outcomes and outperform a common clinical benchmark. We presume that the classifier's access to a condensed representation of the full 3D shape information as opposed to a single value to coarsely approximate said 3D shape is the key reason for this result. This allows the classifier to take into account finer and more localized patterns without getting overwhelmed by too much information, as the complex 3D shape has already been sensibly encoded by the mesh VAE's encoder in a non-linear way. We note, however, that the objective of this experiment was not to achieve the best possible classification performance but rather to generally showcase the utility of the mesh VAE's latent space representation for this task. Hence, we achieve good results without any specific classification loss term during network training but relying only on general encodings of shape variability. Such a multi-task learning approach would likely have improved the separability of the latent space to further differentiate between MACE and no MACE cases, while maintaining a high degree of interpretability. This high degree of extensibility is a key advantage to the presented mesh VAE approach which we aim to explore further in future work.

### 4.5. Network architecture and training

In general, the positive results obtained by the mesh VAE in the previously discussed experiments demonstrate that both its architectural design, loss function, and training procedure were adequately chosen for effective cardiac anatomy modeling with 3D surface mesh data. The graph convolutional layers combined with the mesh downsampling and upsampling operations enable multi-scale feature learning in a hierarchical setup that successfully considers both global and local aspects of cardiac shape variability, which is important to its many possible clinical and research applications. While this is in principle similar to conventional convolution and pooling operations on voxelgrid data, we find that these achieve higher reconstruction errors than a geometric deep learning architecture that is specifically designed to process triangular surface mesh data. In addition, the mesh VAE achieves this outperformance in terms of accuracy while using only about 4% of the number of trainable parameters. This significantly reduces the training time and memory requirements of the algorithm and allowed us to train and evaluate our deep learning models on a standard CPU as opposed to a GPU which is typically required for the 3D voxelgrid VAE. At the same time, the mesh VAE allows for anatomical shapes to be represented as triangular surface meshes which reduces the required data storage costs considerably compared to voxelgrids despite not losing any anatomical information. Furthermore, meshes allow for a continuous encoding of vertex coordinates as opposed to the discretization needed to store similar data in grid-based formats. This sets an upper bound on the possible data resolution due to limited available memory which in turns affects the quality of the anatomical representation.

The choice of VAE framework also allows for straightforward ways to include other metadata, such as patient characteristics or acquisition conditions, into the network as conditional inputs in addition to the anatomical shape information (36, 67). These can, for example, be included as per-vertex features in combination with the coordinate values, or concatenated to the latent space vector or intermediate layers of the network (36, 67, 68). Such an extension enables subpopulation-specific cardiac anatomy modeling while still using a single network and dataset. This is in contrast to PCA, which would need to be applied separately for each subpopulation and would hence only be able to use smaller partitions of the original dataset without making use of synergies across the subpopulations. In order to then achieve the same performance, more data would likely be necessary, whose acquisition is particularly costly in case of medical images.

Regarding the mesh VAE's training procedure, we find that setting the weighting parameter β in the loss function to a suitable value for the given dataset is important to find the right balance between reconstruction and latent space quality and enable effective cardiac anatomy modeling. In our experiments, prioritizing reconstruction quality and using a monotonic annealing schedule resulted in the best overall performance which is in line with previous applications of the β-VAE framework to 3D shape modeling (36, 55). In addition, the mesh VAE is also highly flexible and can work with a variety of different input modalities and reconstruction pipelines, as long as vertex correspondence between meshes in the dataset is ensured. Its architecture also allows for the easy integration of other network components (e.g., as a separate encoder or decoder branch) and multiple different objectives (e.g., cardiac disease classification) in a multi-task learning setting without loss of interpretability. This is akin to grid-based VAEs and therefore creates the possibility of further improvements in similar use cases (27–34).

### 4.6. Limitations

The proposed shape modeling approach also comes with some limitations. All meshes in the input dataset need to exhibit vertex-to-vertex correspondence between each other. As this needs to be established in the preprocessing steps, it limits the method's flexibility and increases its complexity. While this is a common requirement for most shape modeling approaches, including PCA, it is in contrast to voxelgrid-based (28) and point cloud-based shape modeling approaches (36), where such point correspondence is not strictly needed. As a deep learning approach, the mesh VAE requires 2–3 h of CPU training time in our current setup before the population-wide shape variability is accurately captured and follow-up tasks can be performed. This is contrasted with traditional machine learning approaches for the same purpose, such as PCA, which are typically faster in determining their respective data transformation parameters. However, as mentioned in Section 4.5, the mesh VAE still compares favorably in terms of memory footprint and training time to other deep learning approaches based on voxelgrid or point cloud processing.

Furthermore, we have only investigated shape variability in the left ventricle and at the ED and ES phases in this work. In addition, we have trained separate models for ED and ES data which likely results in limited per-subject correspondence between ED and ES meshes in the generated populations. However, we believe that the presented approach can be extended to other cardiac chambers and to other phases of the cardiac cycle, including a combined multi-temporal modeling setup, which we plan to explore in future work. This could be achieved by introducing conditional inputs into various parts of the current architecture that control the cardiac phases to be modeled. Alternatively, separate phase-specific encoder-decoder blocks could be used with a shared latent space to capture multiple cardiac phases at once. This would then likely enable the shape analysis and virtual population generation of paired ED and ES heart meshes. We also note that errors introduced in the preprocessing steps (e.g., MRI segmentation, 3D surface reconstruction) of our shape modeling pipeline affect the results of both the mesh VAE and its follow-up tasks presented in this work. In particular, the reconstruction step does not take into account the information of long-axis slices of the cine MRI acquisition, leading to possible inaccuracies in the basal and apical areas of the 3D heart mesh. While the 3D surface reconstruction step explicitly tries to correct for slice misalignment due to respiratory motion during image acquisition, there are likely still some smaller errors present in the resulting meshes. However, we find that the mesh VAE can successfully process such cases and is often even able to remove unnatural curvatures of the anatomical surface in its reconstructed outputs.

We have also only evaluated the mesh VAE on post-MI subjects and not on a purely healthy cohort. Specifically regarding the MACE classification experiment, we did not consider any patient metadata that would likely help to further improve the results (e.g., sex, age). However, we note that the objective of this work was not to achieve the best possible performance in a single one of the presented tasks but rather to showcase the versatility and applicability of the mesh VAE as a novel approach to 3D cardiac anatomy modeling.

## 5. Conclusion

To conclude, we have presented the mesh VAE as a novel approach to 3D cardiac anatomy modeling that can be directly applied to surface meshes of the heart in an efficient manner. We have demonstrated its ability to accurately capture complex 3D cardiac shapes at both ends of the cardiac cycle while using low-dimensional and easily interpretable latent space representations. The mesh VAE also compares favorably to voxelgrid-based deep learning approaches in terms of both accuracy and memory requirements. Furthermore, we have shown its utility for two exemplary applications, namely the generation of realistic virtual population cohorts of 3D cardiac anatomies and the prediction of MACE outcomes in post-MI patients.

## Data availability statement

The datasets presented in this article are not publicly available. Generated virtual data can be made available upon reasonable request. Requests to access the datasets should be directed to VG, vicente.grau@eng.ox.ac.uk.

## Ethics statement

The studies involving human participants were reviewed and approved by TATORT-NSTEMI trial: Ethical Committee at the University of Leipzig and all Local Ethical Committees of the participating sites; AIDA-STEMI trial: Ethics Committee of the National Regulatory Authorities and the participating centers. The patients/participants provided their written informed consent to participate in this study.

## Author contributions

VG and MB conceptualized and designed the study. AS, TL, TS, SB, HT, and IE acquired the input dataset. JC, AB, EZ, PL, AB-O, and MB implemented and applied the preprocessing steps. MB developed the deep learning methods, conducted the experiments, and created a first draft of the manuscript. VG supervised the work. All authors revised and approved the final version of the manuscript.
